# Longitudinal measurement invariance of the Dutch Outcome Questionnaire-45 in a clinical sample

**DOI:** 10.1007/s11136-017-1500-1

**Published:** 2017-02-13

**Authors:** Ruslan Jabrayilov, Wilco H. M. Emons, Kim de Jong, Klaas Sijtsma

**Affiliations:** 1 University Medical Center Groningen, University of Groningen, Hanzeplein 1, 9713 GZ Groningen, The Netherlands; 2 Department of Methodology and Statistics, Tilburg School of Social and Behavioral Sciences (TSB), Tilburg University, PO Box 90153, 5000 LE Tilburg, The Netherlands; 3 Institute of Psychology, Clinical Psychology Unit, Universiteit Leiden, PO Box 9555, 2300 RB Leiden, The Netherlands

**Keywords:** Change assessment, Item response theory, Longitudinal measurement invariance, Outcome measurement, Outcome Questionnaire 45 (OQ-45), Response shift

## Abstract

**Purpose:**

In the absence of measurement invariance across measurement occasions, change scores based on pretest–posttest measurements may be inaccurate representations of real change on the latent variable. In this study, we examined whether measurement invariance held in the Dutch version of Outcome Questionnaire-45 (OQ-45).

**Method:**

Using secondary data analysis of a sample of *N* = 540 Dutch outpatients, we tested the stability of the factorial structure (gamma change) and the metric and scalar invariance (beta change) across pretest and posttest measurements using a combination of factor analysis and item response theory methodology.

**Results:**

Results revealed a stable factorial structure from pretest to posttest and minor violations of metric invariance for two items in the Dutch OQ-45.

**Conclusion:**

Even though for two items the assumption of invariance was violated, results suggest that the effects of these violations on practical change assessment using the OQ-45 were negligible.

## Introduction

Assessing psychotherapy outcomes typically involves taking into account the difference between pretherapy and posttherapy scores on a self-report questionnaire, thus assuming that the test has invariant measurement properties across time. The assumption of temporal measurement invariance is violated when the relationship between the item responses and the underlying latent variable changes over time. As a result, the meaning of change scores is ambiguous because it is unclear whether observed change is due to real change on the latent variable or caused by other, irrelevant factors [1, 2]. Research has also shown that questionnaires failing to demonstrate measurement invariance over time tend to have a poor reliability and poor predictive validity (e.g., [3, 4]).

Golembiewski, Billingsley, and Yeager [5] distinguished two types of change in the relationship between latent variable and responses, both resulting in violations of longitudinal measurement invariance. The first type of change occurs when the respondents recalibrate the item response options at posttest. For example, at posttest a patient may perceive the response option “often being unhappy” to represent levels of unhappiness that are different than levels perceived at pretest. Such subjective recalibration of response options invalidates change measurement based on pretest and posttest scores, because measurements at both occasions are subjectively normed by different behavioral anchors. As a result, observed change may be spuriously large even though actual change is small, and vice versa. This type of change is known as beta change [5]. Absence of beta change suggests metric and scalar invariance [6].

The second type of change between pretest and posttest measures is called gamma change [5], and occurs when respondents’ fundamental understanding and definition of a latent attribute changes between measurement occasions. For example, respondents may perceive symptoms of distress as an indication of anxiety at pretest but the therapy they undergo may have focused on recognizing different types of stressors, thus leading the measurement away from anxiety at posttest. Gamma change can hinder meaningful change assessment, because pretest and posttest scores represent conceptually different latent attributes. Hence, for valid use of outcome measures in psychotherapy it is important that both beta and gamma change are ruled out, so that observed-score change only reflects real change. In quality of life research, occurrence of beta or gamma change is interpreted as evidence of response shift [7–9].

In this study, we investigated if there is evidence of gamma or beta change in the Dutch Outcome Questionnaire-45 (OQ-45 [10, 11]) across repeated administrations within treated patients and if so, what the consequences are for practical change assessment. The OQ-45 is a widely used self-report questionnaire for monitoring patient functioning [12] throughout treatment in three different functional domains [11]. These functional domains are related to the symptoms of distress experienced on intrapersonal (e.g., ‘I feel no interest in things’), interpersonal (‘I am concerned about family troubles’) and societal levels (e.g., ‘I feel stressed at work/school’). However, only when OQ-45 measurements are invariant across measurement occasions can observed change on the OQ-45 be attributed to real change in these functional domains.

## Method

### Participants and data

A secondary data analysis was conducted using data from $N=540$ outpatients [8, 13]. Data were collected at three treatment departments within two medium-sized mental healthcare institutions in the Netherlands (see [13]). A wide range of psychiatric disorders are treated at these institutions, including disorders related to mood, anxiety, adjustment and personality. The patients in the sample all underwent therapy by a trained therapist and on average completed the OQ-45 3.78 times (min: once, max: 13 times, median: 3 times) throughout treatment. Eighty patients completed the OQ-45 only once and were excluded from the analyses. Ten respondents had more than five missing item scores on either the pretest or posttest; these patients were also excluded from the sample, which resulted in a final data set of 450 patients and a negligible percentage (0.17%) of incidental missing item scores. A statistical models were fitted using maximum-likelihood estimation, which can adequately handle data including missing values. For the remaining 450 patients we used as pretest and posttest scores the data from the first administration and the very last administration, respectively. Using for each patient the measurements that were most distant in time, response shifts—if present—were given maximum opportunity to affect the response process, thus rendering their discovery most likely. Second, for the final measurement the patient knows that the treatment is going to be completed and this awareness may also induce response shift. Table 1 shows several background characteristics of the sample; for more details see [13].

**Table 1:** Sample characteristics of the total sample and the analyzed sample

Background variable	Total sample *N* = 540	In analysis *N* = 450
Background characteristics		
Gender (female)	63.1%	61.7%
Mean age (SD)		
At pretest	37.6 (11.6)	36.8 (11.8)
At posttest	–	37.3 (11.9)
Education (# cases)	(*n* = 448)	(*n* = 285)
Low	129 (27.7%)	61 (21.4%)
Medium	239 (51.4%)	170 (59.6%)
High	80 (17.2%)	54 (18.9%)
OQ45 scores at pretest		
Symptom distress (SD)	45.4 (16.0)	47.7 (14.9)
Interpersonal relations (IR)	15.6 (6.5)	15.8 (6.2)
Social role (SR)	12.1 (5.0)	12.6 (5.2)
Anxiety and somatic distress (ASD)	24.5 (9.4)	25.8 (8.7)
Total score OQ-45	73.2 (23.9)	76.1 (22.3)
OQ-45 posttest and change scores: mean score posttest (SD); % improvement; % deterioration^a^		
Symptom distress (SD)	–	41.3 (16.5); 35.3%; 6.7%
Interpersonal relations (IR)	–	14.8 (6.7); 7.6%; 4.7%
Social role (SR)	–	11.5 (5.0); 7.1%; 3.1%
Anxiety and somatic distress (ASD)	–	22.4 (9.4); 19.6%; 3.8%
Total score OQ-45	–	67.6 (25.1); 36.0%; 7.8%

^a^Improvement and deterioration were defined using criteria for minimum-score difference for reliable change, as reported in the Dutch manual [40]. Only changes in excess of this criterion are considered reliable. The criteria were: 10 score points for Symptom Distress (SD); 8 score points for Interpersonal Relations (IR); 8 score points for Social Role; 9 score points for Anxiety and Somatic Distress (ASD); and 14 score points for total OQ-45 scores

### The Outcome Questionnaire-45 (OQ-45)

The OQ-45 [10, 11] contains 45 Likert items with response options with scores ranging from 0 (never) to 4 (almost always). Together the items comprise three subscales, which are the Symptom Distress (SD; 25 items; example items include “I feel fearful”, and “I feel worthless”) subscale, which taps symptoms of the most common types of psychological distress encountered in practice, such as depression and anxiety; the Interpersonal Relations (IR; 11 items; example items include “I am concerned about my family troubles” and “I have an unfulfilling sex life”) subscale, which measures problems encountered in interpersonal relations; and the Social Role (SR; 9 items; example items include “I feel stressed at school/work” and “I enjoy my spare time”) subscale, which taps distress on a broader social level including distress encountered at work, during education, and during leisure activities.

Two remarks with respect to the OQ-45 are in order. First, it has been shown that the hypothesized three-factor structure of the OQ-45 proposed by Lambert and colleagues [10] is not always replicable (e.g., [14–17]). In addition, De Jong et al. [11] have identified an additional subscale containing 12 items from the SD subscale in the Dutch OQ-45. These 12 items measure symptoms of distress related exclusively to anxiety and its physical manifestations. The authors have named this subscale Anxiety and Somatic Distress (ASD), but the clinical relevance of ASD as a separate scale of patient functioning is not yet evident. Therefore, we used both De Jong’s [11] hypothesized factorial structure and the empirical structure resulting from our sample to study the OQ-45 for beta and gamma change.

Second, previous studies [11, 18] with respect to the psychometric properties of the Dutch OQ-45 revealed four items (i.e., items 11, 12, 26, and 32), which were problematic because of poor fit with the other items in the corresponding subscales. Response shifts cannot be validly detected for these items because they hardly share any variance with other items and their poor fit within the scale may also confound other results. Therefore, these four items were excluded from the analyses. After the exclusion of the problematic items, 24 items remained in the SD, 10 in the IR and 7 in the SR subscales.

### Data analysis strategy

Beta and gamma change have to be assessed sequentially; that is, first, one has to ascertain that the same latent attribute is being measured at both measurement occasions (i.e., no gamma change, but maybe beta change) before proceeding to investigating possible beta change [19]. Therefore, we first concentrate on gamma change and then on beta change.

*Gamma change* To assess gamma change one has to investigate whether the number of factors has changed and if not, whether for a fixed number of factors the pattern of fixed and free factor loadings has changed from pretest to posttest [2, 20, 21]. To accomplish this goal, we first fitted a series of factor models, starting with the one-factor model, then proceeding with the two-factor model, the three-factor model, and so on. No restrictions were imposed on the loadings. The model with the smallest number of factors that adequately fitted the data was retained for further analysis. Next, gamma change was assessed by comparing the patterns of loadings and cross loadings between pretest and posttest in the best-fitting-factor model; that is, we tested for so-called configural invariance [22]. Gamma change was inferred when either (1) a particular item had the highest loading on different factors at pretest and posttest, or (2) the number of factors on which the items had substantial loadings changed across pretest and posttest. All factor models were fitted on the polychoric correlation matrix, using MPlus5.0 [23] and weighted least squares means-adjusted (WLSM) estimation. Factor analysis of polychoric correlation matrices avoids finding spurious difficulty factors [24].

*Beta change* Beta change was assessed for each of the four OQ-45 subscales (i.e., SD, IR, SR, and ASD) separately within the framework of unidimensional IRT [25]. Unidimensional IRT models can be conceived as non-linear factor models for categorical indicators. In particular, we used the graded response model (GRM; [26]), which is suitable for modeling data obtained by means of Likert items, as in the OQ-45. Let $\theta$ denote the latent variable. The GRM assumes unidimensionality, local independence, and a logistic (i.e., S-shaped) relationship between $\theta$ and the cumulative response probabilities. In particular, for each item this logistic function is parameterized by one slope parameter ($a$) and $M$ threshold ($b$) parameters, where $M$ equals the number of response categories minus 1; that is, for a 5-category Likert item, $M=4$ (the reason is that the probability of having a score of at least 0, that is, any score, equals 1, which is a trivial result). The slope parameter expresses how well an item distinguishes low and high $\theta$ values, and thus how strongly observed scores are associated with the latent variable. The threshold parameter ${{b}_{m}}$ ($m=1,\ldots ,4$ for OQ-45 Likert items) denotes the location on the $\theta$-scale where the probability of obtaining score *m* or higher equals 0.50. Different items usually have different $a$ and $b$ parameters. Beta change amounts to change in the item parameters, either $a$, $b$, or both, between pretest and posttest, provided that items are calibrated on the same scale at pretest and posttest. The GRM assumptions of unidimensionality and local independence were evaluated using the residual correlations under the 1-factor model. The assumptions are considered valid if the residual correlations do not exceed 0.15 [27].

For testing beta change, we used likelihood-ratio tests (LRT; e.g., [28]) that are available in FlexMIRT [29]. The LRT compares the likelihood of two nested models, one model that assumes that both the $a$ and $b$ parameters are equal at pretest and posttest (i.e., restricted model of no beta change) and one in which the $a$ and $b$ parameters for one or more items are freely estimated at pretest and posttest (i.e., the general model suggesting beta change). A significant LRT means that the fit of the restricted model is significantly worse than the fit of the general model, thus suggesting that either the slopes or the thresholds changed from pretest to posttest.

*Comparison of factor and IRT approaches* Theoretically, assessing gamma change is also possible within an IRT framework. In fact, assuming multivariate normally distributed latent variables, the factor model of polychoric correlations and the multidimensional GRM are equivalent [30], but the models are estimated differently [31]. Parameters of the factor model are estimated from the bivariate associations, which is the limited information approach. Parameter estimation in multidimensional IRT is based on the likelihood of the response patterns, thus including all high-order associations, and is a full-information approach. Research [31] showed that both approaches yield accurate estimates, but full information approaches may run into computational problems. Therefore, we chose to factorize the polychoric correlations using the limited-information approach for examining gamma change.

Beta change can also be assessed by means of factor analysis. It is tested whether factor intercepts and/or factor loadings changed between pretest and posttest (e.g., [2, 32]). Factor loadings are conceptually equivalent to slope ($a$) parameters in IRT. However, the interpretation of the item intercept in linear factor models is somewhat different from the interpretation of the $b~$ parameters in IRT models. The intercept in a factor analysis can be conceived as the overall item difficulty, whereas the $b$ parameters in the GRM define the probability to score in a particular category or higher and, thus, describe the item-difficulty at the level of the response categories. In practice, item intercepts in factor analysis are rarely utilized for assessing beta change [13]. More importantly, because the GRM has *M* location parameters per item, IRT is better able to exhibit subtle forms of beta change when violations of measurement invariance pertain only to some categories but not to all. Such beta changes may not be visible as change in the intercepts in factor models, because the intercept summarizes information that IRT divides across the *M* threshold parameters, thus allowing to reveal nuances the intercept hides.

## Results

### Gamma change

The three-factor model was the most parsimonious model which had acceptable fit according to the CFI and TLI (both >0.95, Table 2), and moderate fit according to the RMSEA (0.083, Table 2). Comparison of the three- and the four-factor models showed only minor differences in model fit, both at pretest and posttest. These results suggest that a three-factor model provides an adequate description of the data structure at both time points. These results are consistent with previous studies [10, 11]. Therefore, we proceeded with the three-factor model.

**Table 2:** Fit statistics of one- through four-factor models

# Factors	Goodness-of-fit statistics			
	RMSEA	CFI	TLI	SRMR
Pretest data				
1	0.133	0.861	0.854	0.097
2	0.111	0.908	0.898	0.079
3	0.083	0.952	0.943	0.058
4	0.075	0.963	0.954	0.051
Posttest data				
1	0.158	0.902	0.897	0.096
2	0.127	0.940	0.933	0.075
3	0.093	0.969	0.964	0.053
4	0.084	0.976	0.971	0.046

Results without items 11, 12, 26, 32

To compare the pattern of factor loadings under the three-factor model between pretest and posttest, we first fitted the three-factor model in which the items were allowed to load on all three factors. However, because the sample size was small relative to the number of parameters to be estimated, and because of the many cross loadings, the factorial solution was expected to be unstable, rendering its generalizability limited. Therefore, for both pretest and posttest data we re-fitted the three-factor model in which all non-significant cross-loadings were fixed to the items without cross-loading were used to identify the scale. The resulting model fitted well (pretest: CFI = 0.956, TLI = 0.951, RMSEA = 0.078; posttest: CFI = 0.974, TLI = 0.971, RMSEA = 0.086). The pattern of factor loadings that emerged in the restricted three-factor model was different from the original three-factor model proposed by Lambert et al. [10, 33]. Their three-factor model was also fitted to the data, but this model showed poor fit both at pretest and posttest (TLI and CFI <0.95 and RMSEA >0.10 at both pretest and posttest). To avoid drawing conclusions from a poorly fitting model, we proceeded with the restricted three-factor model that emerged in the current sample.

Closer inspection of the factor-loading pattern under the restricted three-factor model showed a consistent configural pattern of low and high loadings at pretest and posttest (Table 3). Only for item 3 factor loadings were inconsistent. The item loaded on two factors, both at pretest and posttest, but the factor on which the item had the highest loading differed between pretest and posttest. The standardized loadings on the posttest were generally a little higher; differences ranged from 0.02 to 0.15. This trend may be explained by an increase of the factor variance at posttest due to inter-individual differences in the magnitude of change after therapy. To conclude, the results suggest that even though the loadings were unequal (suggesting possible beta change), the pattern of cross-loadings was comparable between pretest and posttest. Hence, in the Dutch OQ-45 gamma change is absent. However, the factorial structure is inconsistent with theoretical expectations derived from Lambert et al. [10, 33], both at pretest and posttest.

**Table 3:** Factor loadings for the confirmatory three-factor model

Item No.	Content	Hypoth^a^	Pretest			Posttest		
			F1	F2	F3	F1	F2	F3
1	Friendship	IR	**0.59**			**0.65**		
2	Tiredness	SD (ASD)		**0.56**			**0.68**	
3	Interest in things	SD	0.27	**0.40**		0.41	**0.49**	
4	Work/school related stress	SR		0.27	**0.59**			**0.72**
5	Blaming oneself	SD	**0.41**	0.38		0.38	**0.51**	
6	Irritation	SD	0.30	**0.33**	0.24	0.25	**0.48**	
7	Relationship related happiness	IR	0.32			**0.36**		0.31
8	Suicide ideation	SD	0.37	**0.39**		0.32	**0.48**	
9	Feeling weak	SD (ASD)		**0.68**			**0.75**	
10	Feeling fearful	SD (ASD)		**0.74**			**0.80**	
13	General happiness	SD	**0.66**	0.28		**0.66**	0.28	
14	Work/study balance	SR			0.48			**0.60**
15	Self-esteem	SD	**0.47**	0.47		0.49	**0.54**	
16	Family troubles	IR		**0.36**			**0.47**	
17	Sex life	IR	**0.32**			**0.36**		0.28
18	Loneliness	IR	**0.46**	0.30		**0.46**	0.44	
19	Having arguments	IR	**0.27**		0.26	**0.35**		0.25
20	Love by others	IR	**0.71**			**0.82**		
21	Leisure pleasure	SR	**0.51**	0.30		**0.59**	0.24	
22	Concentration	SD		**0.55**			**0.71**	
23	Hopelessness	SD	0.34	**0.51**		0.29	**0.60**	
24	Self-esteem	SD	**0.61**	0.30		**0.64**	0.31	
25	Rumination	SD (ASD)		**0.69**			**0.72**	
27	Pain in stomach	SD (ASD)		**0.43**			**0.53**	
28	Work/study	SR			**0.25**			**0.33**
29	Palpitations	SD (ASD)		**0.60**			**0.65**	
30	Friendship	IR	0.**64**		0.28	**0.51**		
31	Life satisfaction	SD	**0.72**	0.24		**0.72**	0.25	
33	General anxiety	SD (ASD)		**0.64**			**0.74**	
34	Muscle pain	SD (ASD)		**0.40**			**0.55**	
35	Anxiety in public places	SD (ASD)		**0.53**			**0.55**	
36	Nervousness	SD (ASD)		**0.73**			**0.76**	
37	Love-life satisfaction	IR	**0.62**		0.22	**0.72**		
38	Work/school performance	SR		0.24	**0.63**			**0.75**
39	Disagreements	SR			**0.82**			**0.76**
40	Emotional problems	SD		**0.44**			**0.54**	
41	Sleeping problems	SD (ASD)		**0.57**			**0.61**	
42	Distress	SD	0.32	**0.61**		0.32	**0.66**	
43	Relationship	IR	**0.73**			**0.76**		
44	Angriness	SR			**0.69**			**0.61**
45	Headaches	SD (ASD)		**0.46**			**0.56**	

For each item the largest loadings at pretest and posttest are printed in boldface. Cross-loadings significantly smaller than zero (at the 5% level, two-tailed) are not reported.

^a^ *Hypoth* hypothesized three-factor model of Lambert et al. [33]: *SD* symptom distress, (*ASD* anxiety and somatic distress, see De Jong et al. [11]). *IR* interpersonal relations, *SR* social role

### Beta change

For beta change analysis, we adopted the original composition of the SD, IR, and SR subscales [10, 11, 33], but with the exclusion of the four poor fitting items. IRT analyses of the original subscales showed adequate fit of the GRM. In particular, inspection of the residual correlations under the one-factor model revealed a few values in excess of 0.15 [27], suggesting possible local dependencies. Local dependencies may hamper effective IRT modeling, because they may inflate the estimated $a$ parameters. Therefore, for locally dependent item pairs it was tested whether $a$ parameter estimates were significantly biased using the Jackknife Slope Index (JSI; [34]). The JSI is an estimate of the bias due to local independence. None of the JSIs was significantly different from 0 at the 5% level. Therefore, we proceeded assessing beta change at the subscale level, assuming unidimensionality.

The LRT for testing beta change across time requires a subset of time-invariant items, also known as the anchor set, which can be used to account for real change in functioning at pretest and posttest [35]. A commonly used strategy to empirically select the anchor set is scale purification [36]. The purification procedure first takes the whole set of items as the initial anchor set. Each item in the initial anchor set is tested for significant beta change, using the other items as the anchor items. The item showing the largest beta change is removed from the anchor set, thus producing a new initial anchor set containing one item fewer than the previous set. This procedure is repeated until a final set of anchor items is found without items showing significant beta change. To avoid inflated Type I error rate, in each iteration we used a Bonferroni corrected significance level of $~0.05/k$, where $k$ represents the number of tested items.

The scale purification process revealed two items showing potential beta change over time. These were items 38 (“I feel that I am not doing well at work/school”) from the SR subscale, and item 42 (“I feel blue”) from the SD subscale. Final LRTs of these items using purified anchors confirmed significant beta change in either $a$s or $b$s: ${{\chi }^{2}}\left( 5 \right)=18.1$, $p<0.01$ for item 38, and ${{\chi }^{2}}\left( 5 \right)=22.0$, $p<0.01$ for item 42. For item 38, beta change was caused by a change in both the $a$s and $b$s, whereas for item 42, only the $b$s were significantly different between pretest and posttest. Table 4 shows the estimated item parameters for these items at pretest and posttest.

**Table 4:** Estimated item parameters for the graded response model at pretest and posttest for items 38 and 42

Measurement occasion	Estimated item parameters				
	*a*	*b*_*1*_	*b*_*2*_	*b*_*3*_	*b*_*4*_
I feel that I am not doing well at work/school (item 38)					
Pretest	2.14	−0.73	0.08	0.96	1.96
Posttest	3.65	−0.79	0.18	1.03	1.77
I feel blue (item 42)					
Pretest	2.75	−1.31	−0.73	0.37	1.71
Posttest	3.07	−1.60	−0.53	0.62	1.69

**Fig. 1:**
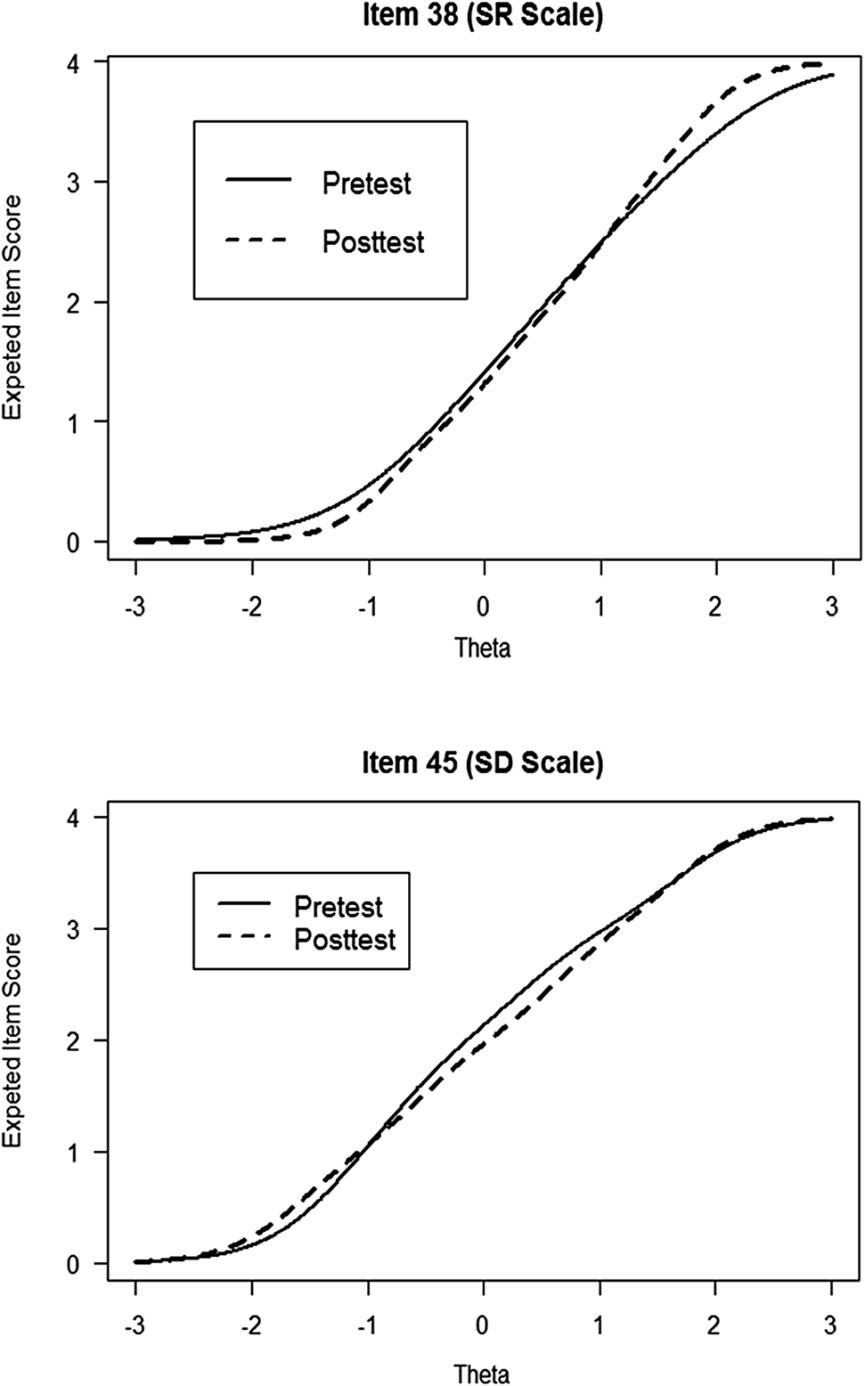
Expected item scores for items 38 and 42 as a function of the latent variable ($\text{ }\!\!\theta\!\!\text{ }$). *SR* social role, *SD* symptom distress

To assess the practical impact of beta change on OQ-45 outcome measurements, for each item we compared between pretest and posttest the relationship between the expected item score and $\theta$ (Fig. 1). The graphs in Fig. 1 suggest that the impact of beta change on practical measurement was minimal. Conditional on $\theta$, the largest difference between the expected items scores at pretest and posttest was 0.27 for item 38, and 0.20 for item 42. This means that on average beta change explained at most a change of 0.27 item-score units. Given that the items are scored on a 5-point scale, we consider a bias of 0.27 to be practically unimportant. Therefore, we concluded that even though items 38 and 42 showed significant beta change between pretest and posttest, the impact of beta change on practical change assessment in the Dutch OQ-45 was negligible.

## Discussion

Response shift involving gamma change or beta change is considered an important threat to the validity of change scores obtained in pretest–posttest designs (e.g., [7–9]). To our knowledge, this study was the first attempt to assess temporal measurement invariance by means of the Dutch OQ-45 in the population of outpatients. Our study provides evidence that despite the beta change in two items the Dutch OQ-45 can be used safely in change assessment based on pretest and posttest scores. Even though we did not find evidence of response shift, more research is needed to draw general conclusions with respect to the absence of beta or gamma change in measurement using the OQ-45.

Two issues to consider with respect to our study are the following. First, given that we did not find gamma or important beta change between the measurements most distant in time, we hypothesize that absence of gamma or beta change also generalizes to the other administrations. Second, the LRT for beta change assumed that there is a set of items that do not show beta change. However, when all items show equal amounts of beta change, the beta change is absorbed in the latent variable distribution and the purification process does not find potentially biased items. Uniform beta change across all items may appear unlikely, but this is an empirical issue that needs further study. Future research may focus on alternative approaches for detecting uniform beta change. An interesting approach may be combining data from a pretest–posttest design with data collected by means of so called ‘then-tests’ methods (e.g., [12, 37]). The idea is that at posttest patients answer some of the questions considering their health status at pretest together with some questions considering their current status.

We did not find gamma change exhibited by a factor structure that was different at pretest and posttest. To conclude, gamma change analyses suggested that the same attribute is being measured at pretest and posttest. However, the factor structure found differed from the hypothesized three-factor solution of Lambert et al. ([10, 33]). It is not clear what explains these inconsistencies, but individuals from different populations may entertain different conceptualizations of items [17]. For example, item 21 (“I enjoy my spare time”) was assigned to the SR scale, but we found a high loading on the factor related to SD. We considered this not as very surprising, because failing to enjoy spare time may be driven by poor social relationships, but also by depressive thoughts and distress. Hence, this item may be indicative both of social role and symptom distress.

In spite of the ambiguous factorial structure and the many cross loadings, the GRM used for the beta change analysis fitted the subscales surprisingly well and all items in the same scale contributed to reliable measurement of the underlying factor. The adequate fit can be explained by the high correlations between the factors and the many cross loadings causing items to fit well within the postulated scales even though factor analysis assigns the item to a different scale. Another issue when using IRT-based LRTs for beta change is the assumption of uncorrelated measurement errors. This assumption, which is technically known as local independence, is restrictive. We notice that local independence is also assumed when testing individual change for significance using the reliable change index [38]. Little research has been done on the presence and explanations of individual-level correlated errors and how such correlations may affect, for example, the power of LRTs or the power to detect individual change. This is also a topic for future research. For assessing gamma change, we estimated the models separately at pretest and posttest, such that correlated errors, if any, did not play a role.

This study focused on evidence of beta or gamma change at the group level. However, evidence of response shift at the group level still leaves open the possibility of response shift in some individual patients. Future research may focus on methods for detecting individual patients showing response shift. One approach could be person-fit analysis [39], which aims at detecting individuals whose response pattern is unlikely given the measurement model. Person fit-analyses has been applied successfully to explain cross-sectional differences in aberrant responding to the Dutch OQ-45 [19]. Future research may consider dedicated person-fit methods for detecting individual response shift.

This study also yielded some interesting results regarding the OQ-45 in general. Support was found for the three factor model, but several items had substantive cross loadings. For example, item 8 (“I have thoughts of ending my life”) and item 18 (“I feel lonely”) seemed to reflect both symptom distress and interpersonal relations. These items may represent general distress which results in a tendency to disengage from social contact, and thus impaired interpersonal relationship. Items with high cross-loadings may be better replaced by items that have a more-specific content. For example, “I feel lonely” could be replaced by the stronger targeted item ‘I have no one with whom I can share my thoughts’ (interpersonal relations). In addition, two social role items (item 19: “I have frequent arguments” and item 28: “I am working/studying less well than I used to”) had no cross-loadings but loaded less than 0.4 on the social role factor. Hence, these items are weak indicators of social role and may need rephrasing or or be removed from the OQ-45.
